# The Immune Landscape of Visceral Adipose Tissue During Obesity and Aging

**DOI:** 10.3389/fendo.2020.00267

**Published:** 2020-05-15

**Authors:** Saad Khan, Yi Tao Chan, Xavier S. Revelo, Daniel A. Winer

**Affiliations:** ^1^Department of Immunology, University of Toronto, Toronto, ON, Canada; ^2^Division of Cellular & Molecular Biology, Diabetes Research Group, Toronto General Hospital Research Institute (TGHRI), University Health Network, Toronto, ON, Canada; ^3^Center for Immunology, University of Minnesota, Minneapolis, MN, United States; ^4^Department of Integrative Biology and Physiology, University of Minnesota, Minneapolis, MN, United States; ^5^Department of Pathology, University Health Network, Toronto, ON, Canada; ^6^Buck Institute for Research on Aging, Novato, CA, United States; ^7^Department of Laboratory Medicine and Pathobiology, University of Toronto, Toronto, ON, Canada

**Keywords:** metabolism, obesity, aging, diabetes, insulin resistance, immunology, immunometabolism, visceral adipose tissue

## Abstract

Obesity and aging represent major health burdens to the global adult population. Both conditions promote the development of associated metabolic diseases such as insulin resistance. The visceral adipose tissue (VAT) is a site that becomes dysfunctional during obesity and aging, and plays a significant role during their pathophysiology. The changes in obese and aging VAT are now recognized to be partly driven by a chronic local inflammatory state, characterized by immune cells that typically adopt an inflammatory phenotype during metabolic disease. Here, we summarize the current knowledge on the immune cell landscape of the VAT during lean, obese, and aged conditions, highlighting their similarities and differences. We also briefly discuss possible linked mechanisms that fuel obesity- and age-associated VAT dysfunction.

## Introduction

Obesity and aging represent two of the largest global health issues of our time. Obesity currently affects over a third of the world's population. Alarmingly, 57.8% of the global adult population are estimated to be overweight or obese by 2030 (1–3). Obesity is part of a condition known as Metabolic Syndrome (MetS), which is defined as a cluster of metabolic risk factors such as abdominal obesity, dyslipidemia, hyperglycemia and insulin resistance (IR) (4). MetS is characterized by a state of low-grade inflammation that is implicated in the development of chronic diseases such as type 2 diabetes (T2D), non-alcoholic fatty liver disease (NAFLD) and cardiovascular disease (CVD) (5–7).

The biological processes of aging and senescence are accelerated during MetS and the risk of developing chronic diseases increases with age (8). In the years to come, aging is expected to represent an enormous challenge for social and health systems. Without adjusting for the rise in longevity, the median age of the world's population is expected to increase from 26.6 to 37.3 years between 2000 and 2050 and to 45.6 years by 2100 (9). This increase in aging will be likely associated with an elevated risk of developing chronic diseases that will aggravate the functional abilities and quality of life of the elderly (10). The changes in the immune system associated with aging are characterized by an imbalance between inflammatory and anti-inflammatory pathways, leading to low-grade inflammation and a greater susceptibility to chronic disease (11).

Obesity and aging contribute to the development of chronic low-grade inflammation in multiple tissues. This low-grade inflammation is recognized as an important factor that promotes downstream consequences of obesity and aging, including the control of whole-body metabolism. During obesity and aging, cells of the innate and adaptive systems accumulate inside the VAT where they alter the local inflammatory environment and impact insulin sensitivity (12). Indeed, the transition from lean to obese or aged states is accompanied by distinct and associated immune cell-driven inflammatory processes. This review will focus on the immune changes that occur in adipose tissue and the means by which immune cells shape the progression of VAT-specific obesity-associated inflammation (OAI) and age-associated inflammation (AAI).

## Vat Plasticity and Remodeling

The adipose tissue is considered an endocrine organ that becomes remodeled during metabolic diseases and is involved in the progression of obesity- and aging-associated IR. There are two distinct types of adipose tissue: white adipose tissue (WAT) and brown adipose tissue (BAT). WAT acts an energy store by accumulating free fatty acids whereas BAT has the capacity of undergoing thermogenesis to dissipate energy (13, 14). WAT can be further divided into two major depots: subcutaneous and visceral. Subcutaneous WAT forms a layer under the skin in the hypodermis while visceral WAT surrounds the inner organs in the abdominal cavity and mediastinum (14, 15). VAT is deposited in certain locations such as the mesenteric fat between the intestines, and the retroperitoneal fat surrounding the kidneys; each VAT store consists of adipocytes and the stromal vascular fraction (SVF) (14). The mesenteric fat pads in mice are the most analogous to human VAT but are not well-studied due to limitations in surgical access (16). Perigonadal fat pads in mice are the most accessible and are used in the majority of mouse VAT studies; however, humans do not have such identical fat depots as mice and as such, these differences should be considered when comparing humans and mice (16). While adipocytes are tightly packed unilocular cells that are supported by a dense network of capillaries, the SVF consists of extra-cellular matrix (ECM) that holds together various cells such as pre-adipocytes, stem cells, fibroblasts, vascular endothelial cells and immune cells (13, 17–19).

During obesity, adipose tissue expansion is characterized by adipocyte hyperplasia (increase in adipocyte numbers) and hypertrophy (increase in adipocyte size), which results in increased adipocyte hypoxia, dysregulation of fatty acid fluxes, increased chemokine secretion, adipocyte cell death, and the recruitment of pro-inflammatory cells (20–23). In turn, pro-inflammatory cytokine release mediated by immune cells induces serine phosphorylation of insulin receptor substrate-1 (IRS-1) leading to local and systemic insulin resistance, which is thought to contribute toward whole body glucose and fatty acid metabolic dysregulation (24, 25) Furthermore, the obese adipose tissue can exhibit a pro-fibrotic phenotype characterized by an increased expression of ECM proteins such as collagen, which can impact adiposity, glucose homeostasis, and susceptibility to metabolic disease (26–29). On the other hand, aging results in an increase in body fat percentage, expansion of the VAT due to a shift in lipid storage from the SAT to the VAT, increased accumulation of senescent cells, and an altered pre-adipocyte cell phenotype (30). During aging, the accumulation of senescent preadipocytes in the adipose tissue leads to increased production of pro-inflammatory cytokines in a process dependent on the JAK pathway (31). It has been hypothesized that an elevated presence of senescent cells and reprogrammed pre-adipocytes in the aged VAT results in the generation of chemokines, pro-inflammatory cytokines, and ECM modifiers which contribute to VAT inflammaging (30).

The innate and adaptive immune systems are now widely accepted as forces that respond to and participate in the remodeling processes taking place in the VAT during metabolic diseases. While inflammatory changes in fat are likely required for proper adipose tissue remodeling and expansion, it is the chronic nature of the inflammation which ultimately drives metabolic disease during obesity and aging (32). In this review, we will discuss the role of immune cells in these two conditions with a focus on their function in the VAT.

## Innate Immune Cells

### Macrophages

#### Homeostasis

Macrophages are a fundamental component of the innate immune system, with the ability to phagocytose harmful pathogens and apoptotic/necrotic cells. Historically, macrophages have been broadly categorized into two categories: the pro-inflammatory “classically activated” M1-like and the anti-inflammatory “alternatively activated” M2-like macrophages. However, macrophages are now thought to exist on a spectrum of functionalities based on their resident and recruited status (33). Macrophages were the first immune cell to be characterized inside VAT during obesity and, until recently, have been the primary focus of most studies. Under homeostatic conditions, the VAT is home to a group of macrophages known as Adipose Tissue Macrophages (ATMs), which represent roughly 5–10% of the stromal vascular fraction, display an M2-like phenotype, depend on the expression of peroxisome proliferator activated receptor γ (PPARγ), and secrete anti-inflammatory IL-10 (34–36). Fate mapping techniques have revealed that not all tissue resident macrophages terminally differentiate from monocyte precursors or emerge from adult hematopoiesis; indeed, some ATMs may develop from bone marrow (BM)-independent progenitors in the embryonic yolk sac and possess the ability to self-renew (37, 38).

#### OAI

During obesity, the population of ATMs within the VAT increases up to 40–50% of the stromal vascular fraction, become metabolically activated, secrete pro-inflammatory cytokines, and engage in “inflammatory cross-talk” with other immune cells, notably CD4+ T cells (34, 35, 39–43). Obesity-associated ATMs accumulate primarily at crown like structures (CLS), characterized by ATMs surrounding dead or dying adipocytes (44). This accumulation of ATMs is the result of increased infiltration due to chemo-attractive gradients and higher proliferation, both partly driven by the monocyte chemoattractant protein 1 (MCP1) (45–47). It is possible that these mechanisms of ATM accumulation are reflective of the presence of distinct self-renewing yolk sac-derived and BM monocyte-derived ATMs. Efforts are in progress to reconcile these observations. Recent work has implicated secreted molecules in skewing ATM population phenotypes. In addition to pro-inflammatory cytokines such as TNFα and IFNγ, the transmembrane activator and calcium modulator and cyclophiliin ligand interactor (TACI) has been implicated in the skewing of macrophages toward an M1-like phenotype. Indeed, ATMs from TACI^−/−^ mice were biased toward an M2-like phenotype and their adoptive transfer into obese mice rescue their dysregulated metabolic parameters (48, 49). Hormones and/or growth factors, such as insulin growth factor 1 (IGF1), can also impact the balance between M1- and M2-like phenotypes in ATMs (50).

However, as mentioned earlier, segregating macrophages into M1-like and M2-like subsets is not reflective of macrophage heterogeneity and efforts are being made to avoid this categorization (51). The markers and phenotypes of VAT monocytes, macrophages and DCs in the context of obesity have been described (52, 53). Further, macrophage classification is moving toward a genomics approach to identification in order to delineate the many heterogeneous cell subsets (54). Conditions during obesity may produce a “metabolically-activated' phenotype in macrophages that is mechanistically distinct from classical activation, which may explain the complexity in macrophage phenotypes seen in mice and humans (55). In light of this consensus, it was recently reported that the obese VAT contains a heterogenous group of macrophages, with BM-derived CD9+ and Ly6C+ ATMs representing the predominant populations (56). CD9+ ATMs exhibit pro-inflammatory gene signatures, localize in the CLS, are lipid-laden, secrete pathogenic exosomes, and induce a pro-inflammatory response in lean adipose tissue upon adoptive transfer (56). In contrast, Ly6C+ ATMs reside outside the CLS, express factors that support vascular development and organization, contain less lipids, and activate gene programs typical of normal adipocyte physiology upon adoptive transfer into lean mice (56). An expansion of the monocyte-derived CD9+ ATM population was recently confirmed via unbiased single-cell RNA sequencing. This population also displays a transcriptional signature associated with lipid metabolism and phagocytosis, consequently being termed lipid-associated macrophages (LAMs) (54). LAMs express Trem2, depletion of which resulted in their loss, decreasing the formation of CLSs observed during diet-induced obesity (54). However, in the absence of Trem2, mice fed a HFD displayed worsened metabolic parameters, implicating a role of these CD9+ Trem2+ LAMs in “buffering” against CLS-associated lipid plaques (54). While an increase in CLS surrounding lipid metabolizing CD9+ ATMs was reported in both of these studies, there is a need to reach a consensus with regards to their inflammatory profiles, their role in VAT inflammation, and whether lipid metabolism can directly regulate the inflammatory output of LAMs. Recent reports also propose the accumulation of sympathetic neuron/nerve-associated macrophages (NAMs) during diet-induced obesity which mediate the clearance of norepinephrine, a catecholamine that has been implicated in lipolysis and fat mass reduction (57).

#### AAI

In aged mice, the proportion of M2-like ATMs reportedly decreases, M1-like macrophages remains unchanged, and CD11c CD206 double negative (DN) ATMs trended to increase, suggesting that aging also skews ATM phenotypes toward a pro-inflammatory phenotype (58). These changes contrast to obesity where a large increase in numbers of VAT macrophages are seen, though aged macrophages still show an overall inflammatory shift. Similar to obesity, aged ATMs display an elevated expression of the chemokine receptor CCR2, possessed enhance secretion of pro-inflammatory cytokines IL-6, MCP-1, and TNFα, and a decrease in expression of PPARγ, which mechanistically accounts for the loss in M2 ATMs. (58). Recent studies propose that although there is a decrease in the proportions of ATMs in aged mice, aged ATMs lack M1- or M2-like polarization and showed a diversity of activation states (59). Furthermore, NAMs are also detected within the aged VAT, which were activated in an NLRP3-dependent manner to regulate lipolysis and fatty acid release through altering catecholamine bioavailability, thereby affecting VAT lipolysis (59). NLRP3 activity was further linked to the production of cytokines IL-1β and IL-18, which contributes to B cell accumulation in the VAT, as discussed below (60). Notably, NLRP3-deficient mice had restored proportions of ATMs which was postulated to reflect an exhausted senescent-like profile driven by chronic activation of the NLRP3 inflammasome (59). It remains to be determined whether levels of CD9+ LAMs and Ly6C+ATMs are altered in the aged VAT; future research in aging should focus on the heterogeneity of the ATM population within the adipose tissue, similar to what has been done for studies in obesity.

### Innate Lymphoid Cells

#### Homeostasis

Innate lymphoid cells (ILCs) play a crucial role in providing defense against a wide array of pathogens such as parasites, microbes, and viruses as well as demonstrating cytotoxic activity toward tumors (61). ILCs are widely categorized into three groups based on their functional differences: group 1 ILCs (ILC-1), group 2 ILCs (ILC-2), and group 3 ILCs (ILC-3). In mice, ILC2 and ILC3 are the most abundant ILC subsets, and often residing in WAT and the intestine, respectively. In humans, however, ILC1 and ILC3 subsets are the predominant ILC subsets which are found in higher frequencies in mucosal and lymphoid sites such as the colon and ileum (62).

ILC-1s are defined by their ability to produce the pro-inflammatory cytokine IFNγ and have been widely studied in natural killer (NK) cells and mixed ILC-1 cells, which develop from different progenitors and possess unique tissue distribution (63). Under homeostatic conditions, ILC-1s are present in lean VAT, where they are resident cells and rely less on infiltration from the periphery, unlike splenic ILC-1s that constantly recirculate (64, 65). Interestingly, compared to their peripheral counterparts, murine NK1.1+ NKp46+ VAT ILC-1s express low levels of Ly49 and consist primarily of three major populations: immature NK (iNK cells), mature NK (mNK) cells, and mixed ILC-1s (64, 65). Parabiosis studies have revealed that while mNK cells recirculate, mixed ILC-1s and iNK cells are mostly resident within the VAT (64, 65). Under homeostatic conditions, VAT ILC-1s were observed to surround ATMs and regulate their numbers by killing both M1-like and M2-like ATMs (65).

ILC-2s are known for producing T helper 2 (Th2) cytokines such as IL-5 and IL-13, and a large body of work conducted on ILC-2s has focused on their role in promoting “beiging” within the adipose tissue (61). Beiging is a process whereby fat-accumulating white adipose tissue is converted into energy-dissipating brown-like adipose tissue (66, 67). Under homeostatic conditions, the presence of ILC-2s in the VAT is maintained by IL-33. ILC-2s have been implicated in promoting the beiging of adipocytes and the accumulation of eosinophils and M2-like macrophages in an IL-4/IL-13-dependent manner (68–70). Additionally, ILC-2s promote beiging of white adipose tissue through the production and release of methionine-enkephalin peptides that upregulate UCP1 expression in adipocytes (71).

ILC-3s contain various populations of RORγt-expressing ILCs that can produce T helper 17 (Th17) cytokines such as IL-17 and IL-22 (61). It remains to be determined whether ILC-3s are found within the adipose tissue, whether they are tissue resident or recruited from the periphery, and finally, what their role is in the context of diet-induced obesity, aging, and chronic inflammation in the VAT.

#### OAI

In mice, during the early stage of diet-induced obesity, there is a transient increase in VAT ILC-1s, although their proportion decreases within the VAT during chronic obesity (64, 65). ILC-1s from chronically obese VAT show a reduced ability to regulate ATMs which may contribute to their uncontrolled expansion (65). Additionally, IFNγ secretion by VAT ILC-1s is also elevated during short-term HFD feeding in an IL-12/STAT-4-dependent manner, which further contributes to the polarization of macrophages to an M1-like phenotype (65). Furthermore, VAT ILC-1s, and potentially their IFNγ, have been shown to promote adipose fibrogenesis by activating pro-inflammatory CD11c^+^ macrophages and the TGF-β1 pathway in adipocytes. These changes may serve to impair adipose function and glycemic tolerance in both mice and humans (72).

The early stage expansion of ILC-1s is primarily a consequence of the recruitment of mNK cells from the periphery followed by a smaller contribution of local proliferation (64, 65). A pathogenic role for the accumulation of mNK cells is likely, as their depletion improved metabolic parameters and decreased macrophage infiltration in obese mice (73–75). During obesity, adipocytes increase their expression of NK cell activating receptor (NCR1) which may be responsible for the expansion of local VAT NK cells and their IFNγ production (74, 76, 77). While increased IFNγ secretion by NK cells drives metabolic dysfunction during early-stage obesity, TNFα secretion by NK cells may play a larger role in driving inflammation and metabolic dysfunction during chronic obesity (78). During chronic obesity, NK cells have been shown to have an impaired capacity to degranulate or produce pro-inflammatory cytokines, which is linked to their uptake of lipids and subsequent metabolic reprogramming (79).

Chronic obesity also promotes the expansion of a distinct IL-6 receptor (IL-6R)- and colony-stimulating factor 1 receptor (csf1r)-expressing myeloid-signature NK cell (myNK) subpopulation in the perigonadal adipose tissue (PGAT) and blood circulation (80). Specific depletion of myNK cells led to reduced inflammation, obesity, and systemic insulin resistance, which could also be recapitulated through abrogation of IL-6 and STAT-3 signaling that is required for the formation of these unique cells (80). Future research should focus on determining the factors responsible for the change in ILC-1 functionality in the transition between early and late chronic obesity. Identifying the factors involved in NK cell recruitment early in obesity is needed as inhibiting NK cell accumulation within the VAT may serve as a therapeutic avenue to prevent elevated IFNγ levels within the adipose tissue; as well, understanding mechanisms to spare NK cells from becoming metabolically dysfunctional due to the lipid-laden adipose environment during chronic obesity.

ILC-2s have been shown to decrease in frequency and numbers in the obese WAT of humans and mice, which may impair the thermogenic ability of fat (68). As such, ILC-2s, along with eosinophils, discussed below, represent potential targets that can be manipulated to promote beiging and thermogenesis of white adipose tissue.

#### AAI

The role of VAT ILCs in aging is largely unknown. As myNK cells are among the cell types that potently respond to IL-6, a cytokine that is elevated in the VAT and circulation during aging, it is possible that aging affects the number and function of these cells (81). However, a large study in humans recently cataloged the different ILC subsets in humans with obesity and age (62). While age showed only marginal mixed up and down correlations of ILC-1/2/3s in abdominal and mesenteric fat, there was a significant correlative decrease in the presence of intestinal ILC-3s with age, highlighting tissue specific changes of these cells during aging (62). More work is needed to tease out how aging impacts VAT ILC accumulation and their inflammatory potential in mice and humans.

### Neutrophils

#### Homeostasis

Neutrophils are responsible for providing protection against pathogens through the release of secretory granules containing a diverse array of antimicrobial proteins and pro-inflammatory mediators (82). Neutrophils can release reactive oxygen species (ROS) and cytokines to kill extracellular bacteria and recruit additional leukocytes to the region of inflammation (82, 83). Neutrophils can also kill extracellular bacteria through the generation of a web of extracellular fibers known as neutrophil extracellular traps (NETs), which are composed of DNA, histones, and antimicrobial proteins (82–84). Much of the work that has been conducted on VAT neutrophils has focused on their role in obesity. Their frequency inside VAT is very low (<1% of non-adipocyte cells) in the lean state and their role in homeostatic functions in fat is unclear (85).

#### OAI

Neutrophils have been shown to enter the adipose tissue during early high fat diet (HFD) feeding in mice. The frequency of VAT neutrophils increase from <1% of non-adipocyte cells in lean mice to ~2% within 1 week after initiating HFD feeding (85). Their crosstalk with adipocytes is sustained through neutrophil CD11b and adipocyte ICAM-1 interactions (85, 86). VAT neutrophils show increased production of the serine protease, elastase, which promotes inflammation in a toll like receptor (TLR)-dependent manner, and deletion of elastase *in vivo* in mice results in improved metabolic parameters (85). It has also been shown that elastase can decrease the amounts of proteins involved in the insulin signaling pathway such as IRS-1 (87, 88). In humans, clinical evidence points toward increased numbers and activation of neutrophils in obese patients. Neutrophils from obese patients possessed enhanced chemotactic activity and produced elevated amounts of superoxide molecules (87, 89, 90).

HFD-fed mice showed increased release and decreased clearance of NETs and increased autoantibodies against nuclear antigens (86). The excess nucleic acids and related protein antigens worsened metabolic parameters through the activation of VAT macrophages and plasmacytoid dendritic cells in the liver through a TLR-dependent manner while treatment of HFD-fed mice with inhibitors against TLR7/9 or NET formation improved metabolic parameters (86). Future work should aim to understand mechanisms and subsequently design therapies that can be used to reduce the accumulation of these cells within the adipose tissue or inhibit their ability to secrete NETs or elastase during obesity and metabolic disease.

#### AAI

There are no data regarding the role for neutrophils in the adipose tissue during the aging process, though few studies have explored the effect of aging in neutrophils. Neutrophils show age-related impairments in phagocytosis, degranulation, ROS generation, migration, and neutrophil microbicidal activity, which can contribute to the poor resolution of infections in the elderly (91–97). Future research should aim to address what factors contribute to the dysregulation of neutrophils in aged individuals, and whether these changes manifest inside fat.

### Dendritic Cells

#### Homeostasis

Dendritic cells (DCs) are considered the bridge between the innate and adaptive immune system due to their antigen presentation role to prime T cells (98). There are two main subsets of DCs that have been well-studied: antigen presenting classical or conventional DCs (cDCs) and plasmacytoid DCs (pDCs) (98). pDCs are significantly less efficient at presenting antigen and stimulating T cells as compared to cDCs but can secrete copious amounts of type 1 interferon (IFN-1) when activated (98). Recently, it was suggested that pDCs emerge from lymphoid progenitors that are distinct from the myeloid lineage and hence share a different ontogeny from cDCs (99).

Two main populations of cDCs are found under homeostatic conditions in murine VAT, namely CD103+ cDC-1s and CD11b+ cDC-2s, both of which promote a tolerogenic, anti-inflammatory environment in the VAT (100). cDC-1s primarily activate the Wnt/β-catenin pathway whereas VAT cDC-2s upregulate the PPARγ pathway. Depletion of β-catenin and PPARγ in VAT cDCs stimulates a pro-inflammatory response in a mouse model of obesity, suggesting a role of these pathways in cDCs in delaying the onset of metabolic disease (100).

#### OAI

Chronic obesity and expansion of the VAT interfere with β-catenin and PPARγ pathways and abrogate the anti-inflammatory function of cDCs, furthering meta-inflammation (100). Earlier studies in humans and mice demonstrated that obesity is associated with an expansion of VAT DCs, mainly cDCs that accumulate in the VAT in a CCR7-dependent and CCR2-independent manner (101, 102). Another study showed that VAT cDCs have the ability to promote pro-inflammatory Th17 responses (53). pDCs have also been implicated in the pathogenesis of VAT meta-inflammation as they are recruited to the tissue due to elevated levels of the adipokine chemerin, and subsequently activated to promote IFN-1 signals in VAT, resulting in the polarization of ATMs to an M1-like state (103). Furthermore, depletion of IFN signaling by genetic deletion of IFNAR or genetic ablation of pDCs resulted in improved metabolic parameters in HFD-fed mice, strongly indicating the role for this subset in contributing to meta-inflammation (104, 105).

#### AAI

Current research on peripheral DCs suggests that aging alters DC function in humans, including defective phagocytosis of antigen, migratory capacity, and enhanced secretion of pro-inflammatory cytokines upon stimulation with TLR agonists (106). While this change in function may contribute to DC mediated inflammatory change inside VAT with age, the roles of cDCs and pDCs in aged VAT remains to be determined.

### Eosinophils

#### Homeostasis

Eosinophils are major producers of IL-4 and IL-13 and play a significant role in host defense, notably against helminth infections (107). Under homeostatic conditions, eosinophils are abundant in the adipose tissue and participate in the beiging process through their production of IL-4 and IL-13 and subsequent activation and accumulation of M2-like ATMs. The activation of M2-like ATMs may be one factor that regulates the expression of tyrosine hydroxylase and production of catecholamines inside VAT, enhancing thermogenesis. However, the details surrounding the cells involved, including NAMs, and the mechanisms of action, such as altering catecholamine bioavailability, require further study (59, 108, 109). Furthermore, IL-5 within the VAT is believed to be responsible for recruiting eosinophils via IL-5 producing cells, which are predominantly ILC-2s (70, 108).

#### OAI

As a consequence of obesity, there is a decline in eosinophils in the VAT; eosinophil-deficient mice are more susceptible to weight gain and show impaired glucose tolerance, greater insulin resistance, and fewer anti-inflammatory M2-like ATMs (108). Treating obese mice with recombinant IL-5 for 8 weeks successfully restored eosinophil numbers within the VAT but did not reduce weight gain, glucose intolerance, insulin resistance, or alter energy expenditure and beiging capacity, implying that rescuing this immune population is not sufficient in the context of VAT dysfunction; thus, the mechanism of eosinophils in regulating VAT health and function therefore appears to be more complex than initially understood and must be further studied (110).

#### AAI

Knowledge on the role of VAT eosinophils during aging is very limited and requires additional work. In mice, aging has been associated with only a modest change in VAT eosinophils, in contrast with obesity where eosinophils decrease in numbers (111). It has also been indicated that aged human eosinophils possess altered degranulation abilities though with regular adhesive and chemotactic properties (112). Aging also has been recently described to prevent formation of cold-induced beige adipocytes in mice and humans and it remains to be determined whether eosinophils are implicated in this defect, along with other beiging-inducing immune cells (112, 113).

### Mast Cells

#### Homeostasis

Mast cells play important roles during allergy and inflammation, and are components of the myeloid cell population in mouse VAT under homeostatic conditions. Mast cells have been shown to facilitate the preadipocyte to adipocyte transition in VAT (114, 115).

#### OAI

The VAT mast cell population increases during obesity, although they initially decrease slightly in the intermediate stages of HFD feeding in WT mice (12 weeks) but rebound in the later stages (114, 116). In humans, mast cell proportions positively correlated with the typical features of expanded obese VAT such as inflammation of endothelium, ATM build-up, and formation of fibrous tissue (117).

Mast cells can contribute to VAT dysfunction through degranulation and release of proteases such as tryptase, chymase, cathepsins, and matrix metalloprotease-9. These enzymes regulate adiponectin action and catabolize ECM collagens and fibronectin to promote adipogenesis and infiltration of pro-inflammatory cells into the tissue (117–120). Furthermore, mast cell-derived IL-6 and IFNγ contribute to adipose tissue cysteine cathepsin expression, which promotes VAT angiogenesis and growth in obese mice (118, 121). Mast cell-derived cathepsins can also degrade adipocyte insulin receptor and the glucose transporter Glut-4, leading to impaired glucose physiology in adipocytes (122).

Earlier reports indicated that mice deficient in mast cells fed a HFD displayed improved metabolic parameters and reduced pro-inflammatory cytokines in the VAT (118). It was further shown that mast cells regulate metabolism through IL-6 and IFNγ (118). Additionally, Kit^W−sh/W−sh^ mice, which are deficient in mast cells, reconstituted with mast cells from *Il6* or *Ifng* knockout mice were protected from metabolic dysregulation (118). However, other models of mast cell deficiency reported no differences in weight gain, glucose physiology, or VAT inflammation (123, 124). The differences observed between studies are likely due to the use of different models of mast cells deficiency such as the Kit^W−sh/W−sh^ mast cell-deficient mice (which are not specific to mast cells), Kit-independent Cpa3^Cre/+^ and *Mcpt5-Cre R-DTA* mice, and mast cell stabilizer disodium cromoglycate treated mice (118, 123, 124).

#### AAI

To our knowledge, no studies have explored the presence of mast cells in the aged VAT and whether they contribute to tissue specific inflammaging. In mice, aging has been shown to promote the degranulation of mast cells upon exposure to prostaglandin E (PGE), which is not observed in mast cells from young animals (125). Furthermore, aged adipocytes secrete more PGE than young adipocytes and thus this warrants the examination of mast cell degranulation within the adipose tissue and the potential role of various proteases released, in the context of inflammaging and age associated IR (126).

### Myeloid Derived Suppressor Cells

#### Homeostasis

Myeloid derived suppressor cells (MDSCs) are a heterogenous population of immature myeloid cells with anti-inflammatory functions including suppression of adaptive immunity, modulation of macrophage cytokine production, and elevated expression of immunosuppressive factors such as arginase 1 (Arg1) and inducible nitric oxide synthase (iNOS) (127). MDSCs tend to typically develop during a variety of inflammatory conditions such as sepsis, cancer, and instances of autoimmunity but little is known about their presence and/or role in the lean adipose tissue.

#### OAI

MDSCs have been shown to increase in the adipose tissue of leptin-deficient obese mice, while transferring MDSCs into obese mice improves parameters associated with metabolic disease (128). Accordingly, tumor-bearing mice fed a HFD display an enhanced accumulation of MDSCs. While MDSCs protected against metabolic disease and VAT inflammation, they have a detrimental effect on tumor progression and overall reduced animal survival time (129). Several factors have been implicated in the increased accumulation of MDSCs, including the adipokine leptin, polyunsaturated fats, and exogenous lipids, which could potentially be harnessed to boost MDSC activity and deter inflammation (129–131).

#### AAI

In mice, MDSCs have been shown to accumulate in the spleen, lymph nodes, and bone marrow of aged mice and also possess greater suppressive activity in T cell proliferation, which is associated with defective PI3K-Akt signaling pathway (132). Furthermore, MDSCs have been linked to limiting B lymphopoiesis during aging, which is driven via their production of IL-1 (133). Whether MDSCs accumulate in the aged VAT to suppress inflammatory cells or to impact insulin resistance remains to be evaluated.

## Adaptive Immune Cells

### B Cells

#### Homeostasis

B cells are a crucial component of the adaptive immune response that achieve their functions via cytokine secretion, antibody secretion, and modulation of the function of other cells (134). The majority of B cells can be categorized into B1 and B2 cells. B1 cells are enriched in the pleural and peritoneal cavities, while B2 cells are found abundantly in secondary lymphoid organs such as the spleen (134). Regulatory B cells (Bregs) are a collection of heterogenous IL-10 producing B cells that can ameliorate inflammation. Inside VAT, a subset of these Bregs are maintained by CXCL12 and free fatty acids and Breg deletion of IL-10 results in aggravated VAT inflammation, insulin resistance, and loss of metabolic homeostasis (135). However, the majority of B cell-derived IL-10 in VAT at steady state is derived from B1 cells, the B cell population that is also predominantly abundant within fat-associated lymphoid clusters in the VAT (136, 137).

#### OAI

Obesity induces an accumulation of total B cells in the VAT, including the proportion and absolute number of class switched mature IgM– IgD– IgG+ B2 cells (138). IgG isolated from obese mice is capable of driving metabolic disease due to their hyposialylated profile which activates the endothelial IgG receptor FCγRIIB (138, 139). In humans, insulin resistance is associated with a distinct profile of IgG autoantibodies, arguing for (self) antigen-specific targets contributing to B cell-mediated insulin resistance (138). A recent paper extended these findings by characterizing self-antigens targeted by IgG inside human adipose tissue (140). Moreover, B cells isolated from obese mice are responsible for modulating T cells within the VAT in an MHC-I/II-dependent manner by priming both CD4+ and CD8+ T cells. Ablation of the antigen-presenting complex from B cells is sufficient to improve metabolic parameters, indicating a role of B-T cognate interactions in the VAT in modulating metabolic disease (138). Obese B cells also secrete elevated levels of pro-inflammatory cytokines and in line with these various observations, both B cell-deficient μMT mice and anti-CD20-treated mice display improved metabolic and inflammatory parameters (138, 141). Notably, the LTB4/LTB4R1 chemokine/receptor axis has been shown to promote the activation and recruitment of VAT B2 cells, highlighting its potential as a target for insulin sensitizing therapies (142). Unlike B2 cells, tolerogenic B1a cells are reduced in frequency during obesity and produce less IL-10 and transferring B1a cells from lean mice into HFD-fed B null mice can improve metabolic parameters through IL-10- and polyclonal IgM-dependent mechanisms (136). Obesity also induces compromised functionality in natural Bregs (135).

Given the potential pathogenicity of B2 cells and beneficial effects of Bregs and B1 cells, targeting B2-driven VAT inflammation while enhancing the anti-inflammatory function of B1/Breg cells would help alleviate obesity-related adipose pathophysiology. For example, one possible B cell targeting therapy involves the use of B-cell Activating Factor (BAFF) antagonists as BAFF deficiency or inhibition ameliorates obesity-induced inflammation in mice (136, 143). Despite the growing interest in VAT B cells, the role of antibody secreting cells (ASCs) in the VAT is unknown. ASCs are terminally differentiated B cells that secrete antibodies and cytokines and can be further categorized into short-lived plasmablasts and long-lived plasma cells (144, 145). While VAT IgG increase during obesity, it is unclear whether this increase is due to a recruitment of long-lived plasma cells from the bone marrow or from other tissue such as intestines (146).

#### AAI

Like obesity, aging is characterized by a similar increase in mature B2 cells in the VAT, as well as plasma IgG as early as 12 months in mice (147). Furthermore, aging is associated with an increase in the expression of the B cell-specific nuclear co-factor Oct coactivator from B cells (OcaB) in the VAT. Depletion of OcaB in mice improved metabolic parameters, prevented the accumulation of B2 cells within the VAT, and decreased circulating levels of IgG2c and pro-inflammatory cytokines (147). An increase in the number of B1a and B1b cells has also been observed in the aged VAT. However, this accumulation was much subtler and the role of these innate B cells in aging requires further examination (147). Interestingly, some B1 cells develop pathological features during aging. Indeed, monocytes can convert omental B1a cells into 4-1BBL-expressing B1a cells which promotes immune activation and insulin resistance (148).

Interestingly, aging drives the formation of a unique circulating B cell subset known as age-associated B cells (ABCs), which are defined by their overexpression of the transcription factor T-bet (149–151). ABCs are further characterized by their ability to respond to nucleic acid antigen, and produce pro-inflammatory TNFα and IgG2c, making them prime candidates for mediating VAT inflammaging and age-associated insulin resistance (152, 153). Some evidence suggests that ABCs may represent either a memory B cell population or an ASC population as they express elevated levels of transcription factors involved in ASC formation, namely Prdm1, Irf4, and Xbp1, as well as expressing the ASC surface marker CD138 (151, 153, 154). Moreover, members of the SWEF family of Rho guanine exchange factor (GEF) proteins have been implicated in regulating the expansion of ABCs through the activity of IRF5 (155). Such ABCs have also been implicated in autoimmune disorders such as lupus in young mice, suggesting that aging does not serve as a prerequisite for their induction. This notion warrants the need to determine whether they are present within the VAT during diet-induced obesity independently of aging (154). Interestingly, ABC expansion is observed more consistently in female mice and it remains to be examined whether sex-specific hormone or other factors are at the root of these differences (149). Recently, another unique population of B cell, aged adipose B cells (AABs), have been shown to accumulate in aged VAT, which are memory-like B cells that expand within the fat-associated lymphoid clusters (FALCs) in aged VAT in an NLRP3 dependent manner; depletion of these cells can improve insulin resistance in mice and reverse age-induced lipolytic dysfunction, and future work will need to assess triggers and targets regulating these cells function during aging (60, 156).

### T Cells

#### Homeostasis

T cells are a major component of the adaptive immune system and can be categorized into various subsets based on their expression of surface markers, the composition of T cell antigen receptors (TCR), and the secretion of different cytokines. T cells with the αβ TCR rearrangement can be categorized according to their expression of CD4 or CD8, with the former being further sub-divided into various subsets based on their effector cytokine profiles including IFNγ-producing T helper 1 (Th1) cells, IL-4-producing Th2 cells, IL-17-producing Th17 cells and IL-10-producing Foxp3+ T regulatory cells (Tregs).

Tregs are highly enriched in the lean adipose tissue, uniquely regulated by PPARγ, and promote skewing of macrophages to an M2-like state (157–160). Furthermore, the accumulation of Tregs depends on antigens presented in an MHC-II manner and the release of soluble mediators, notably IL-33, the majority of which is secreted by mesenchymal stromal cells (161–163). There is a growing appreciation of an evolutionarily conserved requirement for IL-33 in VAT Treg function, as demonstrated by their dependency upon the IL-33 receptor ST2, and downstream transcription factors BATF and IRF4 (164, 165). Interestingly, recent work has shown that sexual dimorphism has also been reported to play a role in VAT Treg function in this axis (166). VAT inflammation is increased with testosterone and limited with estrogen in males. Increased VAT inflammation and male-specific IL-33-producing stromal cells mediate the recruitment and local expansion of Tregs in a BLIMP-1-dependent manner, constituting a male-specific feedback circuit that potentially limits VAT inflammation (166). Research on Th2 cells in the adipose tissue also points toward a protective role, and human VAT is also thought to be enriched in Th2 cells expressing IL-13 under insulin sensitive conditions (157, 167).

#### OAI

Generally, T cells are known to accumulate within the VAT in obese mice and humans. Depleting T cells with an anti-CD3 antibody improves insulin sensitivity and limits VAT meta-inflammation in obese mice (157, 168). CD4+ cells within the obese VAT produce higher amounts of IFNγ than those from lean VAT, indicating an overall Th1 polarization of CD4+ T cells (157, 169, 170). Indeed, IL-12p35^null^ mice, which are deficient in Th1 cells, show improved insulin sensitivity as the main Th1 effector cytokine, IFNγ, has been shown to be directly responsible for affecting insulin signaling, lipid storage, and differentiation of adipocytes via sustained JAK-STAT1 pathway activation (157, 171).

Obesity results in increased accumulation of VAT Th17 cells in an IL-6-dependent manner, although these cells are present at a lower frequency compared with Th1 cells (157, 170). Mechanistically, extracellular ATP acts through the P2X7 receptor pathway promotes a Th17-polarizing microenvironment (172). This was also accompanied by a greater frequency in Th17 cells and higher expression of Th17 markers such as RORC (RORγt in mice), IL-17, and IL-23R in VAT explants from metabolically unhealthy obese donors compared to metabolically healthy obese and lean donors (172, 173). Despite this research, the role of IL-17 in obesity-induced metabolic disease is unclear. Despite reports of increased IL-17 in obese individuals, mouse models have shown that IL-17 deficiency worsens the effects of diet-induced obesity, accelerates adiposity in mice fed a low-fat diet, and elevates circulating leptin levels (172, 174, 175). However, it is important to note that γδ T cells and MAIT cells are also sources of IL-17 and all studies regarding the impact of IL-17 on metabolic disease cannot be attributed to Th17 cells alone (175). More work is required to distinguish and elucidate the cell specific role of IL-17 in the adipose tissue during metabolic disease.

During obesity, adipose tissue Tregs decrease in number and are outweighed by the function of pro-inflammatory T cells (157, 176). As such, rescuing the Treg population may represent a potential therapeutic strategy in improving metabolic parameters during obesity; treatments with PPARγ agonists such as thiazolidinedione, 5-aminosalicylic acid (5ASA) or IL-33 administration *in vivo* were shown to regulate VAT Treg numbers and improve metabolic parameters in obese mouse models (160, 164, 165, 177).

CD8+ T cells, or cytotoxic T lymphocytes (CTLs), increase in the VAT during obesity and have an enhanced capacity to secrete IFNγ (167, 178). Despite no differences in body weight, obese mice deficient in CD8+ T cells display improved glucose tolerance and insulin sensitivity, suggesting a pathogenic role for CD8+ T cells in impairing metabolic health (105, 178). The cytotoxic activity of CD8+ T cells has been demonstrated to be regulated by perforin, as perforin-deficient CD8+ T cells show an elevated proliferative and inflammatory capacity. Transfer of these perforin-deficient CD8+ T cells into CD8-deficient mice significantly worsened metabolic parameters compared to those transferred with perforin-sufficient CD8+ T cells (75).

Several outstanding questions that remain to be explored include the mechanisms by which co-stimulatory molecules induce T cell pathogenicity during obesity, as well as how T cell metabolism is altered during adipose tissue inflammation. The 4-1BB-4-1BBL checkpoint pathway has been reported to be important in Th1-skewing of CD4+ cells and enhancing CD8+ T cell proliferation (179, 180). In obese mice, there is an increase in the expression of both the receptor and ligand; mice deficient in 4-1BB are reportedly protected from HFD-induced metabolic dysregulation and adipose macrophage and T cell infiltration (181, 182). Thus, it would be possible to target pro-inflammatory T cell function via blockade of this pathway. Recently, our group has reported that insulin receptor signaling modulates T cell inflammatory function and subset skewing via the control of intrinsic cellular metabolism, though it remains to be determined whether insulin signaling in VAT T cells is impacted during chronic obesity (183). Finally, it will be important to identify which antigens are targeted by T cells within the VAT. Indeed, a large majority of T cells in the VAT are effector memory T cells that exhibit a markedly restricted TCR diversity (157, 184). This, along with the presence of pathogenic IgG autoantibodies produced by B cells, suggests that there may potentially be specific target antigens within the VAT that drives an antigen-specific T cell response (140, 185). Whether these antigens are bystanders or true drivers of disease remain to be seen.

#### AAI

Within the aged VAT, similar to obesity, there is an expansion of various T cell populations including CD8+ T cells and CD4+ T cells. However, unlike obesity, Treg numbers increase in VAT with age (58, 111). Age-dependent accumulation of CD8+ T cells appears to be dependent on biological sex as aged CD8+ T cells from the VAT of female mice display an elevated activation status and secrete more pro-inflammatory cytokines than their male counterparts (186). Future work needs to explore the distribution of CD4+ T helper subsets within the VAT micro-environment and what signals are responsible for driving the accumulation of T cells within the aged VAT. Interestingly, Tregs within the VAT are enriched in aged male mice and selectively depleting them improves glucose uptake and age-associated insulin resistance (111), though the mechanisms are unclear. It should be noted that aged females have lower levels of VAT Tregs compared to male counterparts and the mechanism driving this difference is poorly understood (186), though recent work in describing sex differences in VAT Tregs may provide insight to these differences (166). Finally, further work should explore the role of the IL-33-Treg axis in the context of healthy aging, as well as determine whether dysfunctional stromal cell activity contributes toward the expansion of VAT Tregs during aging.

### Innate Like T Cells

#### Homeostasis

There is an emerging appreciation for the role of innate-like T (ILT) cells that possess innate mechanisms to respond rapidly to stress and pathogens while concurrently expressing antigen receptors reminiscent of adaptive immunity (187). There are three main subsets of ILT cells that we will be discussing: natural killer T (NKT) cells, γδ T cells, and mucosal-associated invariant T (MAIT) cells.

NKT cells are a group of ILT cells that are activated upon recognizing glycolipid antigens presented on the non-classical MHC-I-like protein, CD1d, and can be further categorized into two subtypes based on their TCR diversity: type 1 or invariant NKT (iNKT) cells, which express an invariant TCRα chain and limited numbers of TCRβ chains, and type 2 or diverse NKT (dNKT) cells, which show more diverse usage of TCRα and β chains (188, 189). The VAT is enriched with iNKT cells which help maintain the adipose tissue microenvironment under homeostatic conditions by promoting IL-4-dependent M2-like macrophage polarization and sustaining Treg activity in an IL-10/IL-2-dependent manner; indeed, depletion of CD1d in adipocytes worsened inflammation and insulin resistance (190–192). Surprisingly, VAT iNKT cells in mice constitute a specialized tissue resident subset that express the transcription factor E4BP4 and lack PLZF expression, unlike circulating iNKT, and produce IL-2 and IL-10 to regulate immune homeostasis within the VAT (193, 194).

γδ T cells also possess innate-like features that allow for their activation upon recognition of conserved stress-induced ligands and subsequent rapid secretion of cytokines such as IFNγ, TNFα, and IL-17A (195). In lean wild type mice, adipose tissue resident PLZF+ γδ T cells were shown to produce TNFα and IL-17A which together promoted IL-33 production by adipose stromal cells (196). IL-33 production induced by γδ T cells was shown to promote thermogenesis as mice lacking γδ T cells or IL-17A showed impaired adipocyte UCP1 expression and thermoregulation (196).

Mucosal associated invariant T (MAIT) cells are found primarily in peripheral blood, the intestinal mucosa, and the liver and are evolutionary restricted by the MHC-related molecule 1 (MR1) (197). Upon stimulation, human peripheral MAIT cells produce IFNγ, TNFα, IL-17, IL-2, and granzyme B, whereas mouse spleen MAIT produce higher levels of IL-17 and lower levels of IL-10, IFNγ, and TNFα (198–202). In humans, adipose tissue MAIT cells but not peripheral blood MAIT cells produce more IL-10 than IL-17, highlighting site-specific differences in MAIT function (202). Although the role of MAIT cells inside VAT is poorly understood, they are potent secretors of IFNγ and IL-17 and warrant further investigation under homeostatic conditions (197).

#### OAI

During obesity, there is reportedly a decrease in the numbers of iNKT cells; furthermore, CD1d^−/−^ mice deficient in iNKT cells display an insulin resistant phenotype even in the absence of a high fat diet and adipose inflammation, providing evidence for their protective function in the VAT during obesity (190, 203, 204). Conversely, in a Vα14 transgenic mouse model, which has elevated levels of iNKT cells, having increased iNKT cells on a Ldlr^−/−^ background resulted in worsened metabolic parameters when mice were placed on an obesogenic diet. These findings suggest that all iNKT cells might not be outright protective, but rather the reduction in iNKT cells might be a compensatory mechanism in response to obesity (205). This observation could also be explained by the differential roles between peripheral and local iNKT subsets in maintaining tissue homeostasis. Further, most of these studies did not assess the role of type 2 dNKT cells as CD1d^−/−^ mice lack both NKT subsets. dNKT cells have been shown to play a protective role during obesity as adoptive transfer of dNKT cells into obese mice improved weight loss and glucose homeostasis (206). Future studies should aim to use newly developed mouse strains to better address the specific roles of each NKT subsets in obesity (207). The observation that VAT PLZF+ γδ T cells promote the IL-33-Treg axis and thermogenesis under homeostatic conditions warrants the need for studies to explore the functions of PLZF+ γδ T cells in obese adipose tissue, whether they are lost during obesity, and whether IL-17 secretion by γδ T cells is impaired during obesity (196).

Patients with T2D and/or obesity show decreased frequency of peripheral MAIT cells but increased frequency in adipose tissue MAIT cells (202, 208, 209). Further, MAIT cells in both the adipose tissue and periphery from obese patients produced higher levels of IL-17 and reduced levels of IL-10, different from the lean state, suggesting a potential role for MAIT cells in obesity-associated inflammation (210). The mechanisms underlying the alterations in MAIT cell numbers, activation, and IL-17 production need to be further elucidated, with a need to determine the ontogeny of adipose tissue MAIT cells and their role in the adipose environment during obesity.

#### AAI

Only a few studies have explored the role of ILT cells within the aged VAT. In mice, NKT cell numbers were increased 2- to 3-fold in the secondary lymphoid organs of aged mice compared to young mice, suggesting that a similar increase or accumulation of peripheral PLZF+ NKT cells might be seen within the VAT (211). However, it remains to be determined how the population of adipose resident E4BP4+ iNKT cells are affected and whether the distribution of peripheral vs. resident NKT cells is shifted (193). Similar to obesity, it has also been suggested that peripheral MAIT cells are reduced in aged individuals and future studies need to determine whether they are being recruited to metabolic tissues such as the liver and/or VAT (212).

## Concluding Remarks

**Figure 1:**
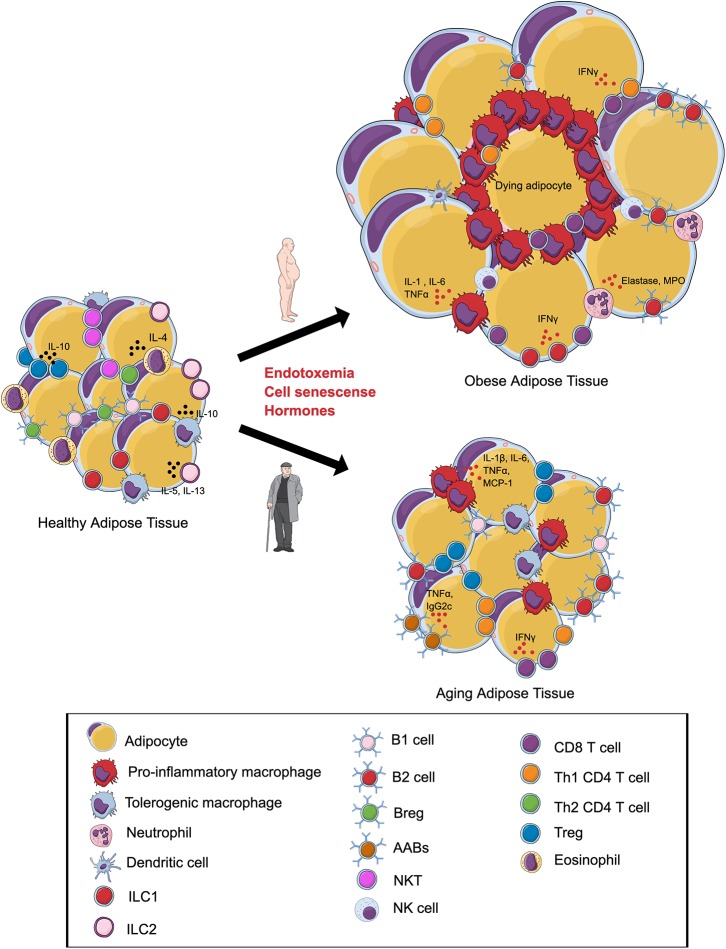
Alterations in the visceral adipose tissue (VAT) immune cells during obesity and aging. In lean VAT, homeostasis is maintained via the secretion of anti-inflammatory cytokines by regulatory T cells (Tregs), T helper 2 (Th2) cells, tolerogenic macrophages, group 1 innate lymphoid cells (ILC1s), ILC2s, regulatory B cells (Bregs), B1 cells, and eosinophils. **(Top)** Obesity induces an expansion in adipocyte size and promotes a shift in the phenotype of local immune cells toward a pro-inflammatory state with increases in pro-inflammatory macrophages, NK Cells, B2 cells, Th1 CD4 cells, CD8+ T cells, and neutrophils. Inflammation of adipose tissue leads to tissue damage, cell death, and metabolic disturbances. **(Bottom)** During aging, the VAT is characterized by alterations in the immune cell environment. Emerging evidence indicates that these changes are associated with a shift in the phenotype of macrophages, expansion of B2 cells, age-associated B cells (AABs), CD8+ T cells and, paradoxically, regulatory T cells (Tregs). Changes in the composition of adipose tissue immune cells during aging may contribute to insulin resistance and ectopic lipid storage. Illustration created in the Mind the Graph platform: www.mindthegraph.com.

Aging and diet-induced obesity present themselves as metabolic diseases that are characterized by an alteration of the VAT immune landscape and display an activation of chronic inflammatory pathways that contribute to insulin resistance and diabetes (Figure 1, Tables 1, 2). There are several overlapping mechanisms of VAT inflammation in obesity and aging that could help direct future research. One common driver of VAT inflammation in both conditions is the altered gut and the resident host microbiota. Obesity is associated with microbial dysbiosis and an overall reduction in bacterial diversity, which imparts features of the metabolic syndrome as the obese phenotype can be transferred from obese humans to mice through transplant of the gut microbiota (213–216). A consequence of microbial dysbiosis is increased intestinal permeability, characterized by leakage of bacterial antigen or their products such as metabolites or pathogen associated molecular patterns including lipopolysaccharide (LPS) across the intestinal epithelial barrier. These products can access metabolic tissues such as the VAT by entering systemic circulation and also being taken up by chylomicrons, which further drives meta-inflammation by activating pro-inflammatory cascades in immune cells via pattern recognition receptor signaling (177, 217–219). Aging is also potentially associated with intestinal microbial dysbiosis that contributes to worsened intestinal permeability, serum endotoxemia and inflammation, which can be recapitulated upon transfer of gut microbiota from aged mice into young germ-free mice, as seen during obesity (219–221). Low grade chronic intestinal inflammation is an early manifestation of obesity which precedes systemic metabolic disease and contributes to worsened barrier function and insulin resistance via secretion of pro-inflammatory cytokines (213). Moreover, an increased flux of bacterial product entering the VAT may be one common mechanism linking aging and obesity related insulin resistance to distinct downstream inflammasome receptors such as the NLRP3 inflammasome, which ultimately represent immune activation in response to gut bacterial products in obesity and aging (60, 156).

**Table 1:** Comparison between innate immune cells in OAI and AAI.

**Innate immune cells**	**Obesity**	**Aging**
Macrophages	↑ in ATM population [H/M] (33–35, 39–43) ↑ pro-inflammatory cytokine release [H/M] (33–35, 39–43) ↑ inflammatory cross-talk [H/M] (33–35, 39–43) ↑ crown-like structures [H/M] (44–47) ↑ in pro-inflammatory CD9+ macrophages (LAMs) [H/M] (54)	↑ pro-inflammatory phenotype [M] (58) ↓ proportion of M2-like ATMs [M] (58) ↔ proportion of M1-like ATMs [M] (58) ↑ proportion of CD11c- CD206- ATMs [M] (58) ↑ expression of CCR2, IL-6, MCP-1, TNFα [M] (58) ↓ expression of PPARγ [M] (58)
**ILCs**		
- ILC-1	↑ proportion in early obesity [H/M] (64, 65) ↓ proportion in chronic obesity [H/M] (64, 65) ↑ IFNγ and TNFα secretion [H/M] (65, 74, 76, 77) ↑ mNK recruitment and expansion [H/M] (64, 65) ↓ in NK degranulation and pro-inflammatory cytokine production during chronic obesity [H/M] (79) ↑ myNK expansion in chronic obesity [H/M] (80)	↔ proportion [H] (62)
- ILC-2	↓ frequency in WAT [H/M] (68)	↔ proportion [H] (62)
- ILC-3	x	↔ proportion [H] (62)
Neutrophils	↑ frequency [H/M] (85, 89) ↑ activation [H/M] (85, 89) ↑ production of elastase [M] (85) ↑ release and ↓ clearance of NETs [M] (86)	x
**DCs**		
- cDCs	↓ anti-inflammatory profile [M] (10) ↑ accumulation of VAT cDCs [H/M] (101, 102) ↑ promotion of Th17 cell responses [H/M] (53)	x
- pDCs	↑ recruitment to VAT [H/M] (103–105) ↑ IFN-1 signaling in VAT and polarization of ATMs to M1-like state [H/M] (103–105)	x
Eosinophils	↓ eosinophils in VAT [M] (108)	↔↓ eosinophils in VAT [M] (111)
Mast Cells	↑ mast cells in VAT [H/M] (114, 116, 117) ↑ inflammation of VAT endothelium, ATM accumulation, formation of fibrous tissue [H] (117)	x
MDSCs	↑ MDSCs in VAT [M] (128) ↑ protection against metabolic disease [M] (129)	x

*A table of summary for the changes in innate immune cell populations specifically within the adipose tissue in obesity and aging*.

*“x” indicates studies are lacking*.

↑ *indicates increased*, ↓ *indicates decreased, and* ↔ *indicates no change or no consensus*.

*[H/M] indicates data seen in human and/or mouse studies*.

**Table 2:** Comparison between adaptive immune cells in OAI and AAI.

**Adaptive immune cells**	**Obesity**	**Aging**
**B cells**		
- B2	↑ accumulation of B cells (138) - ↑ class switched mature IgG+ B2 cells [M] (138) - Distinct IgG autoantibodies [H/M] (138, 141)- ↑ antigen presentation to T cells [M] (138) - ↑ pro-inflammatory cytokines [M] (138, 141)	↑ mature B2 in VAT [M] (147) ↑ circulating IgG levels [M] (147) ↑ OcaB expression in VAT [M] (147)
- B1	↓ frequency in B1a cells [M] (136) ↓ IL-10 production [M] (136) ↓ Breg functionality [M] (135)	↔↑ numbers of B1a and B1b [M] (147) ↑ accumulation of 4-1BBL^+^ B1a cells [H/M] (148)
- ABCs	Unclear if present in obesity independent of aging (154)	↑ in aging [H/M] (149–153) ↑ in females [M] (149) ↑ accumulation of aged adipose B cells (AABs) [M] (60, 156)
T cells	↑ in VAT [H/M] (157, 168–170)	x
CD4+	↑ in VAT [H/M] (157, 168–170)	↑ in VAT [M] (58)
- Th1	↑ Th1 polarization in CD4+ T cells [H/M] (157, 169, 170) ↑ production of IFNγ [H/M] (157, 169, 170)	x
- Th17	↑ accumulation in VAT [H/M] (157, 170, 172, 173) ↑ expression of Th17 markers (RORC/RORγt, IL-17, IL-23R) [H/M] (172, 173)	x
- Treg	↓ population in VAT [H/M] (157, 176)	↑ in VAT [M] (58, 111) ↑ enrichment in males [M] (111, 166, 186)
CD8+	↑ population in VAT [H/M] (167, 178) ↑ IFNγ secretion [H/M] (167, 178)	↑ in VAT [M] (58, 111) ↑ activation and pro-inflammatory cytokine secretion in females [M] (186)
**ILTs**		
- iNKT	↓ population in VAT [H/M] (190, 203, 204)	x
- dNKT	↑ protection against metabolic disease [M] (206)	x
- γδ T cells	x	x
- MAIT cells	↑ frequency [H] (202, 208, 209) ↑ production of IL-17 [H] (210) ↓ production of IL-10 [H] (210)	x

*A table of summary for the changes in adaptive immune cell populations specifically within the adipose tissue in obesity and aging*.

*“x” indicates studies are lacking*.

↑ *indicates increased*, ↓ *indicates decreased, and* ↔ *indicates no change or no consensus*.

*[H/M] indicates data seen in human and/or mouse studies*.

Another shared factor that could potentially link obesity and age driven VAT inflammation is cellular senescence. Senescence is regarded as a hallmark of aging, and metabolically active senescent cells secrete a variety of cytokines and chemokines, in an NFKB dependent manner, which further drives local inflammation (222, 223). Senescent cells have been demonstrated to also accumulate within the VAT during obesity, and their clearance is associated with improved metabolic parameters and decreased macrophage homing (224). Interestingly, within the VAT, CD4+ T cells adopt a senescent phenotype which drives VAT inflammation and insulin resistance in an osteopontin dependent manner (225). A combination of cell senescence and inflammatory cell death within the fat might represent a source of shared antigenic targets between obesity and aging, which likely underpins a mechanism fueling local immune cells.

Finally, both obesity and aging are associated with changes in the levels of hormones that could impact immune cell function in the VAT. For instance, leptin and insulin typically rise with obesity and aging. Leptin metabolically activates T cells and skews them toward a Th1 cytokine secretion profile, promotes pro-inflammatory cytokine secretion by circulating monocytes, and enhances expression of perforin in NK cells (226–229). Insulin has been shown to facilitate T cell glycolytic programming and IFNγ mediated effector functions (183). The interactions of these hormones and adipokines in an environment of increasing cell senescence and bacterial products likely represents additional common drivers between aging and obesity related VAT inflammation.

Given these potential underlying common drivers of obesity- and aging-related IR, it will be important to understand the mechanistic differences between both conditions. For instance, why do VAT Tregs function differently with age compared to obesity? What are the underlying reasons behind the differences in VAT macrophage numbers? It will also be important to tease out the differences in inflammatory responses between mouse models and humans. In particular, do aged humans show similar changes to VAT immune cells as seen in aged mice? Understanding the differences and similarities between VAT immune populations with age and obesity will help identify unifying pathophysiological root causes of the associated metabolic disease.

Over the past 20 years, our knowledge about the immune landscape of the VAT has grown from the simple identification of the role of cytokines and macrophages, to include adaptive immunity, and lately most known immune cell populations. The level of cellular characterization will continue to improve to further refine these populations using single-cell genomics and advanced cytometry methods. Such analyses will ultimately yield new insights into disease pathogenesis and may lead to new therapies to combat obesity and aging related metabolic disease.

## Author Contributions

SK, YC, XR, and DW contributed to the design and writing of the manuscript and the generation of the figures.

## Conflict of Interest

The authors declare that the research was conducted in the absence of any commercial or financial relationships that could be construed as a potential conflict of interest.
